# Genómica en parasitología: avances y desafíos

**DOI:** 10.7705/biomedica.7746

**Published:** 2024-11-06

**Authors:** Concepción Puerta B.

**Affiliations:** 1 Grupo de Enfermedades Infecciosas, Facultad de Ciencias, Pontificia Universidad Javeriana, Bogotá, D. C., Colombia Pontificia Universidad Javeriana Pontificia Universidad Javeriana Bogotá, D. C. Colombia

Los parásitos han convivido y evolucionado con los seres humanos a lo largo de los años, afinando sus elegantes mecanismos de regulación de la expresión génica para escapar de la respuesta inmunológica de su huésped, reproducirse y perpetuarse.

Las ciencias ómicas han tenido un profundo impacto, tanto en el avance del conocimiento de la relación huésped-parásito, como en la lucha contra las enfermedades causadas por parásitos. Esto, gracias a que hemos pasado de estudiar unos pocos genes individuales a analizar genomas, e incluso transcriptomas, proteomas o metabolomas completos en condiciones específicas de estudio. Este es el caso del estudio del proteoma de *Leishmania braziliensis* en condiciones de crecimiento normales y de estrés para identificar proteínas de unión al ARN (*RNA-Binding Proteins,* RBP) potencialmente asociadas con la regulación de la proteína de choque térmico de 70 kDa (HSP70) implicada en diversos procesos celulares determinantes para la supervivencia del parásito ^1^. En este estudio, no solo se develó la participación de más de 50 proteínas en dicho proceso, sino que se identificaron nuevas proteínas del parásito capaces de unirse al ARN y participar en la regulación génica ^2^^,^^3^, lo cual ilustra desde entonces la potencialidad y versatilidad de este tipo de abordajes.

Históricamente, tras el desarrollo del proyecto del genoma humano, surgió la inquietud de descifrar los genomas de los microorganismos patógenos que afectan a los humanos, al igual que los de sus vectores biológicos; el de *Plasmodium falciparum* fue uno de los primeros proyectos relacionados, publicado en el 2002. Los genomas se han constituido en el pilar para el desarrollo de las demás disciplinas ómicas y, actualmente, dadas las ventajas de los métodos de secuenciación de nueva generación (*Next Generation Sequencing*, NGS), se busca ya no solo conocer el genoma de un individuo representante de la especie, sino el genoma de toda una población. Nace así la genómica de poblaciones, la cual permite estudios tan importantes como el de las dinámicas evolutivas de la especie y su impacto en las medidas de intervención.

Además de dar a conocer la estructura de los genomas y la identidad de sus genes, los proyectos genómicos han sido cruciales en el desarrollo de métodos diagnósticos, ya que han sido la base para el diseño de múltiples formatos de la reacción en cadena de la polimerasa (PCR) a partir de segmentos de ADN o ARN presentes únicamente en el organismo que se quiere detectar, pero ausentes en el huésped o en otras especies cercanas de parásitos que circulan en las mismas regiones endémicas. Ejemplos de esto son las pruebas desarrolladas para el diagnóstico de la enfermedad de Chagas en los formatos de PCR en tiempo real ^4^ y de *Loop-Mediated Isothermal Amplification* (LAMP) ^5^.

Por su parte, la genómica comparativa generó nuevas dinámicas y la comparación de especies cercanas ha permitido, entre otros aspectos, diferenciar especies patógenas de no patógenas, identificar factores de virulencia y esclarecer procesos evolutivos como el estatus de especie o subespecie de algunos parásitos. Swapna y Parkinson, en su revisión de 2017, ilustraron el impacto de la genómica comparativa de los protozoos parásitos del filo *Apicomplexa*^6^.

Los estudios de transcriptomas, proteomas y metabolomas han sido sumamente valiosos para identificar las moléculas y vías relacionadas con una función en particular. Inicialmente, se abordó la búsqueda de genes que se expresan preferiblemente en un estadio parasitario o en una condición específica de espacio o tiempo. Más adelante, se desarrollaron aproximaciones para identificar vías metabólicas específicas o, incluso, proteínas que interactúan con determinados blancos, dando lugar a términos como *kinome* e *interactome*, entre muchos otros. En el estudio de Sabalette *et al*. ^7^, se comparó el transcriptoma del parásito *Trypanosoma cruzi* en condiciones de aumento y de disminución de la expresión del gen que codifica para la proteína TcUBP1 (*Regulatory U-rich RNA Binding Protein 1*). Los resultados revelaron los genes cuya expresión es controlada por la TcUBP1 en el paso de diferenciación de la etapa de epimastigote a la infectiva del parásito. Estos son hallazgos que pueden orientar el estudio de nuevos blancos contra la enfermedad de Chagas.

El análisis de los genes y de las vías metabólicas implicadas en la resistencia a fármacos también se ha nutrido de las ciencias ómicas. En su publicación de 2023, Pandit *et al*. ^8^ revisaron los mecanismos de resistencia desarrollados por *P. falciparum* frente a la artemisinina y encontraron perfiles metabólicos distintos entre los parásitos resistentes y los sensibles. La metabolómica también ha servido para investigar los mecanismos de muerte parasitaria inducidos por fármacos o productos naturales, como es el caso del trabajo de Pardo-Rodríguez *et al*. ^9^, en el cual, mediante distintos enfoques metabolómicos, se reveló que la apoptosis era el mecanismo principal de muerte inducida en *T. cruzi* por un extracto de hexano de la planta *Clethra fimbriata* que también tiene potencial inmunomodulador.

Como sucede con otras infecciones, el reto es lograr la erradicación o, al menos, el control de las enfermedades parasitarias. No obstante, en cuanto a esto, debemos recordar que, al estar los parásitos constituidos por células eucariotas, al igual que el ser humano, lograr diseñar fármacos ideales con poca o ninguna reacción secundaria, es una tarea bastante ardua y difícil. Varios caminos se han recorrido en este esfuerzo; el primero de ellos es, quizá, la exploración de vías metabólicas únicas de los parásitos, ausentes en el humano; o, en caso de estar presentes en ambos organismos, que la estructura o función de las moléculas fuese lo suficientemente diferente como para lograr un ataque selectivo. Actualmente, laboratorios de distintas partes del mundo, incluso de países endémicos para las parasitosis, se han unido en aras de realizar proyectos de alto rendimiento para identificar -entre miles de millones de moléculas- blancos potenciales que luego serían estudiados y validados mediante genómica funcional ^10^.

Particularmente, considero que un campo inexplorado para el diseño y desarrollo de fármacos antiparasitarios es la regulación de la expresión génica parasitaria. Se sabe que las RBP son capaces de regular la expresión en varios niveles y que una sola puede afectar a varias proteínas, incluso con funciones distintas, lo cual redunda en el bloqueo de diferentes vías parasitarias. Lo anterior puede, por una parte, garantizar la eliminación del parásito y, por otra, dificultar la generación de resistencia a los fármacos empleados. En este sentido, sin duda, las aproximaciones ómicas son fundamentales para abarcar el estudio de diversos blancos. Justamente, Ruiz *et al*. muestran una ruta para identificar las RBP en tripanosomátidos ^11^.

La generación de vacunas antiparasitarias ha sido otro campo difícil de abordar, al que se suman retos como la variabilidad genética de los parásitos, sus finos mecanismos de evasión de la reacción inmunológica, la asociación particular de la respuesta inmunológica protectora con moléculas específicas del complejo mayor de histocompatibilidad y el cambio a lo largo de los años de la conducta humana y su relación con el entorno. Los enfoques ómicos han contribuido también en este aspecto mediante la identificación de blancos potenciales para el desarrollo de vacunas y la construcción de rutas bioinformáticas basadas en los datos de los proyectos ómicos para el desarrollo de vacunas, como lo proponen Michel-Todó *et al*. ^12^ en el caso de la enfermedad de Chagas.

El estudio de la respuesta inmunológica del huésped es otro campo fértil e imprescindible que también se alimenta de las ciencias ómicas y que puede contribuir a: (I) el hallazgo de biomarcadores de seguimiento, pronóstico o eficacia de intervenciones en pacientes; (II) el desarrollo de herramientas terapéuticas, como anticuerpos dirigidos contra los receptores de agotamiento de los linfocitos T, y (III), el desarrollo de vacunas antiparasitarias con múltiples epítopes; estas propuestas se revisan en el estudio de Puerta *et al*. ^13^. En el trabajo de Ros-Lucas *et al*. ^14^, por ejemplo, se ilustra cómo, mediante transcriptómica comparativa de células mononucleares de sangre periférica de pacientes con enfermedad de Chagas tratados y no tratados -asintomáticos y sintomáticos- versus las de controles sanos no infectados, es posible identificar moléculas y vías relacionadas con la respuesta inmunológica que pueden constituir biomarcadores de progresión o respuesta al tratamiento.

La regulación epigenética en la dupla huésped-parásito también ha sido objeto de innumerables estudios ómicos con importantes aportes. Particularmente, en parásitos intracelulares, la combinación de distintas disciplinas ómicas con otras herramientas ha venido esclareciendo cómo los protozoarios intracelulares manipulan a su favor el control epigenético y transcripcional de sus células huésped. Así, en el trabajo de Lecoeur *et al*. ^15^, se muestra cómo la leishmania modifica la regulación epigenética y transcripcional de las células dendríticas y los macrófagos, para promover la expresión de un fenotipo antiinflamatorio que favorece el establecimiento de la infección.

En general, es importante subrayar dos características fundamentales que acompañan los enfoques ómicos: la colaboración mundial interdisciplinaria, multidisciplinaria y transdisciplinaria, y la gran cantidad de información que produce cada estudio. La colaboración científica nace, entonces, de la complejidad de los retos aquí señalados y de cómo cada científico puede aportar desde su disciplina, visión y experiencia. Por otro lado, la enorme cantidad de datos que surge de estos proyectos constituye un desafío por sí misma, ya que más que saber seleccionarlos, depurarlos, normalizarlos, clasificarlos, integrarlos, sistematizarlos y analizarlos, se debe aprender a hacer las preguntas pertinentes y, por supuesto, disponer de la capacidad técnica y tecnológica que todo ello supone. Lo cierto es que, en esta multiplicidad de bases de datos asociadas con pruebas biológicas, publicados normalmente en revistas especializadas en la divulgación de datos, se dispone de un gran recurso para el avance de la ciencia. Por ejemplo, en el estudio de Ruiz *et al*. ^16^, se evidencian las proteínas asociadas con los ARN mensajeros de LYT1 de *T. cruzi*, un factor de virulencia de este parásito.

Mi visión del futuro de las ciencias ómicas se entrelaza con la ciencia de datos, la medicina traslacional, la epidemiología y la salud pública, de forma que todos estos hallazgos lleguen directamente al paciente y a la población en general. Espero que, por ejemplo, la farmacogenómica, la nutrigenómica y la microbiogenómica, entre otras ómicas, sean las herramientas de rutina de los profesionales de las ciencias del laboratorio clínico, cuyos resultados orienten el estudio y tratamiento personalizados, así como el diseño de medidas poblacionales de intervención ajustadas al momento de la evaluación.
